# Hypercholesterolemia promotes early renal dysfunction in apolipoprotein E-deficient mice

**DOI:** 10.1186/1476-511X-10-220

**Published:** 2011-11-26

**Authors:** Camille M Balarini, Mariana ZT Oliveira, Thiago MC Pereira, Nyam F Silva, Elisardo C Vasquez, Silvana S Meyrelles, Agata L Gava

**Affiliations:** 1 Laboratory of Transgenes and Cardiovascular Control, Physiological Sciences Graduate Program, Federal University of Espirito Santo, Vitoria, ES, Brazil; 2 Laboratory of Neuromorphology, Department of Morphology, Federal University of Espirito Santo, Vitoria, ES, Brazil; 3 Research Center, Emescam College of Health Sciences, Vitoria, ES, Brazil; 4 Biotechnology Graduate Program, Federal University of Espirito Santo, Vitoria, ES, Brazil

**Keywords:** Renal Function, Aging, Hypercholesterolemia

## Abstract

**Background:**

Aging and dyslipidemia are processes which can lead to deleterious consequences to renal function. Therefore, the aim of this study was to determine the effects of both hypercholesterolemia and aging on renal function in mice.

**Methods:**

Male hypercholesterolemic apolipoprotein E-deficient mice (ApoE, n = 13) and age-matched C57BL/6 control mice (C57, n = 15) were studied at 2 (young) and 8 (adult) month-old. At each time point, animals were placed in metabolic cages for 24 hours to urine volume and urinary creatinine quantification. Blood samples were collected for serum cholesterol, urea and creatinine measurements. Glomerular filtration rate (GFR) was estimated through creatinine clearance determination. Mesangial expansion was evaluated by Periodic Acid Schiff staining, renal fibrosis was determined through Masson's trichrome staining and neuronal nitric oxide synthase (nNOS) expression in the kidney was performed by Western Blotting. To statistical analysis two-way ANOVA followed by Fisher's *post hoc *test was used.

**Results:**

Total plasma cholesterol was increased about 5-fold in ApoE mice at both time points compared to C57 animals. At 2-month-old, GFR was already markedly reduced in ApoE compared to C57 mice (187 ± 28 vs 358 ± 92 μL/min, p < 0.05). Adult C57 (-77%) and ApoE (-50%) mice also presented a significant reduction of GFR. In addition, serum urea was significantly increased in young ApoE animals compared to C57 mice (11 ± 1.3 vs 7 ± 0.9 mmol/L, p < 0.01). A significant mesangial expansion was observed at 2-month old ApoE mice compared to C57 mice (35 ± 0.6 vs 30 ± 0.9%, respectively, p < 0.05), which was aggravated at 8-month old animals (40 ± 3 and 35 ± 3%, respectively). Tubulointersticial fibrosis was augmented at both young (17 ± 2%, p < 0.05) and adult (20 ± 1%, p < 0.05) ApoE mice compared to respective C57 age controls (8 ± 1 and 12 ± 2%, respectively). The expression of nNOS was markedly reduced in a time-dependent manner in both strains.

**Conclusions:**

These data show that both hypercholesterolemia and aging contribute to the loss of renal function in mice.

## Background

Cardiovascular morbidity, which includes dyslipidemia among the most striking risk factors, is age-related and represents the leading cause of mortality in occidental population [1]. Genetically predisposing to hypercholesterolemia usually involves alterations in lipoprotein transport and metabolism, leading to atherosclerosis [2-4]. Besides its relation with cardiovascular events, hypercholesterolemia is considered a factor that contributes to renal dysfunction [5] and to worsening the state of patients with previous kidney damage [6]. Patients with dyslipidemia usually show cardiovascular manifestations, however, few studies have focused on the deleterious effects of hypercholesterolemia on the progression of renal disease.

The murine model that lacks the functional gene encoding the apolipoprotein E (ApoE), which spontaneously develops hypercholesterolemia and atherosclerotic lesions similar to those found in human beings [7,8] has greatly contributed to the understanding of these disorders. Apolipoprotein E is a constituent of VLDL synthesized in the liver and other tissues and serves as a high-affinity ligand for apolipoprotein E receptors, and thus is responsible for the cellular uptake of lipoprotein particles [3]. Although dyslipidemia and aging are known to be risk factors for renal diseases, there is a lack of studies concerning the effects of these conditions together in the ApoE mouse.

In the present study we aim to evaluate the effects of both hypercholesterolemia and aging on renal function in the murine model of spontaneous atherosclerosis compared to the normocholesterolemic C57BL/6 (C57) mouse. Our data show that hypercholesterolemia promotes early renal dysfunction in mice and aging contributes to the worsening of this condition. The mechanisms underlying this dysfunction may include mesangial expansion, increased renal fibrosis and reduced expression of neuronal isoform of nitric oxide synthase (nNOS).

## Results

Table 1 summarizes the results of metabolic parameters and serum cholesterol levels in ApoE and age-matched C57 mice. Body weight, chow and water intake and urine production were similar in young ApoE and C57 mice. In adult mice it was observed a significant decrease in chow intake and a significant increase in body weight in both ApoE and C57 groups. Water intake and urine production did not change over the course of aging in both groups. As expected, both young (2-month-old) and adult (8-month-old) ApoE mice presented an augmentation about 5-fold in serum cholesterol levels when compared with age-matched C57.

**Table 1:** Metabolic parameters and plasma cholesterol in young (2-month-old) and adult (8-month-old) C57 and ApoE mice.

Parameters	Young		Adult	
	
	C57 (7)	ApoE (6)	C57 (8)	ApoE (6)
Body Weight (g)	25.6 ± 0.4	24.8 ± 1.0	29.1 ± 1.2^††^	28.1 ± 0.8^†^
Chow Intake (g/24 h)	3.7 ± 0.4	4.0 ± 0.2	2.2 ± 0.2^††^	2.8 ± 0.3^††^
Water Intake (mL/24 h)	8.2 ± 1.6	7.6 ± 0.8	6.3 ± 0.9	5.4 ± 0.6
Urine Production (mL/24 h)	2.7 ± 0.3	2.6 ± 0.4	2.2 ± 0.4	1.7 ± 0.2
Serum Cholesterol (mmol/L)	2.4 ± 0.1	15.8 ± 2.4**	3.0 ± 0.3	16.5 ± 2.0**

Values indicate means ± SEM of the number of animals in parentheses.

**p < 0.01 vs C57; ^†^p < 0.05 and ^††^p < 0.01 vs 2-month-old (2-way ANOVA).

**Figure 1:**
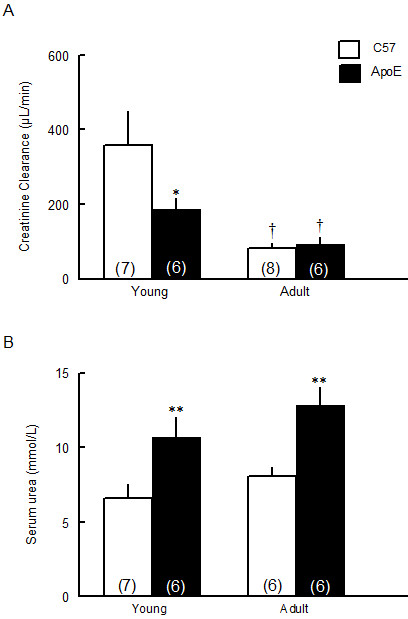
**Renal functional parameters in young and adult C57 and ApoE mice**. Creatinine clearance (A) and serum urea (B) were used to determine the renal function in young (2-month-old) and adult (8-month-old) C57BL/6 (C57) and apolipoprotein E-deficient (ApoE) mice. Values indicate means ± SEM of the number of animals in parentheses. *p < 0.05 and **p < 0.01 vs C57 and ^†^p < 0.05 vs 2-month-old (2-way ANOVA).

The effects of age and hypercholesterolemia on renal function were evaluated through creatinine clearance and serum urea concentration (Figure 1). Young ApoE mice already showed a substantial loss of renal function as indicated by the reduced creatinine clearance compared to age-matched C57 animals (187 ± 28 vs 358 ± 92 μL/min, p < 0.05). In adult mice, creatinine clearance was decreased by about 77% in C57 (81 ± 14 μL/min, p < 0.05) and 50% in ApoE mice (93 ± 18 μL/min, p < 0.05) (Figure 1A). Impaired renal function in young ApoE mice was also indicated by a significant increase in serum urea compared to age-matched C57 animals (10.7 ± 1.3 vs 6.6 ± 0.9 mmol/L, p < 0.01), but this condition was not exacerbated in adult animals (Figure 1B).

The evaluation of glomerular tuft and Bowman's capsule areas and glomeruli number showed that these parameters were not affected by hypercholesterolemia or aging process (data not shown).

**Figure 2:**
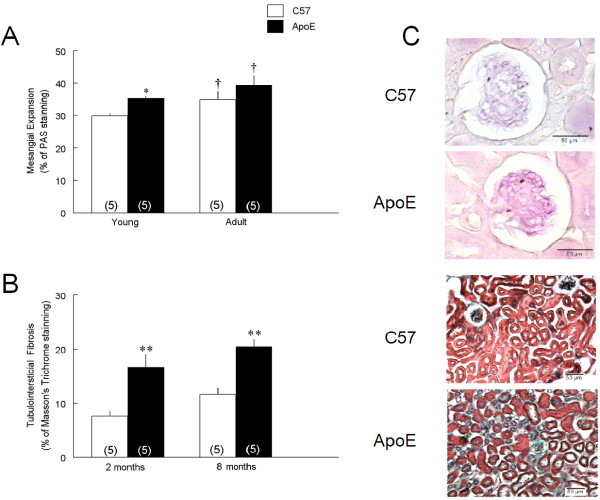
**Histological evaluation in kidneys of young and adult C57 and ApoE mice**. A: the mesangial expansion was evaluated through the percentage of periodic acid-Schiff staining (PAS) in the glomerulus and B: tubulointersticial fibrosis was evaluated by the percentage of Masson's Trichrome staining in the tubular area in young (2-month-old) and adult (8-month-old) C57BL/6 (C57) and apolipoprotein E-deficient (ApoE) mice. C. Typical photomicrograhies showing the increase in mesangial matrix (upper panel, 40 × magnification) and collagen deposition (lower panel, 20 × magnification) in ApoE mice. Values indicate means ± SEM of the number of animals in parentheses. *p < 0.05 and **p < 0.01 vs C57 and ^†^p < 0.05 vs 2-month-old (2-way ANOVA).

Changes in mesangial matrix and tubulointersticial fibrosis indicated by the percentage of glomerular staining with periodic acid-Schiff (A) and Masson's trichrome (B) are shown in Figure 2. Mesangial expansion was observed in young ApoE mice (35 ± 0.6%, p < 0.05) compared to age-matched C57 animals (30 ± 0.9%). Aging process aggravated this condition in both adult strains (40 ± 3% and 35 ± 3%, p < 0.05, respectively). Also, increased collagen deposition was observed in young ApoE mice (16.7 ± 2.3%, p < 0.01) compared with 2-month-old C57 (7.6 ± 1.0%). Aging process did not exacerbate tubulointersicial fibrosis (11.6 ± 1.3 and 20.5 ± 1.3%, respectively). Typical photomicrografies of mesangial expansion and increased collagen deposition are demonstrated in Figure 2C.

**Figure 3:**
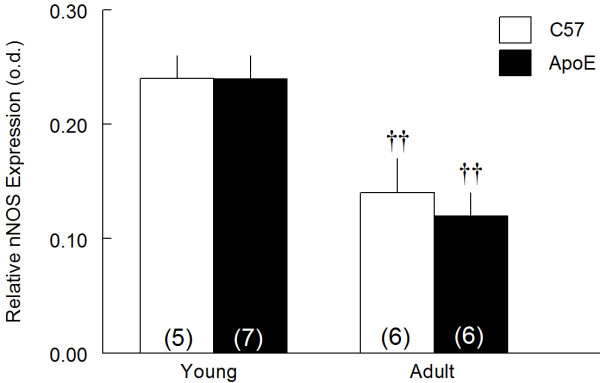
**Effects of hypercholesterolemia and aging on renal nNOS expression**. Renal nNOS expression was assessed by Western Blotting in young (2-month-old) and adult (8-month-old) C57BL/6 (C57) and apolipoprotein E-deficient (ApoE) mice. Values indicate means ± SEM of the number of animals in parentheses. o.d.: optical density ^††^p < 0.01 vs 2-month-old animals (2-way ANOVA).

Figure 3 shows that hypercholesterolemia of young ApoE mice did not affected the renal nNOS expression compared with age-matched C57 animals (0.24 ± 0.02 and 0.24 ± 0.02 o.d.). On the other hand, in adult animals this parameter was reduced in about 50% in both strains (0.12 ± 0.02 and 0.14 ± 0.03, o.d. p < 0.05, respectively).

## Discussion

The present study shows that the hypercholesterolemic ApoE mouse is characterized by an early impairment of renal function when compared with normocholesterolemic C57 animal. The aging process aggravated this condition in ApoE mice and promoted a significant loss of renal function in C57 animals.

The importance of studying a murine model that lacks the functional gene encoding the apolipoprotein E is that it develops spontaneous hypercholesterolemia and atherosclerotic lesions similar to those found in human beings [7,8]. In the present study, young ApoE mice showed significant higher levels of total plasma cholesterol when fed normal chow, in agreement with previous studies [9,10], although they do not show established atherosclerotic lesions [3]. As previously reported by others [3], hypercholesterolemia was not aggravated in adult animals. We also observed a reduced chow intake and increased body weight in both adult C57 and ApoE mice, which are expected metabolic changes in rodents [11,12].

It has already been reported that hypercholesterolemia leads to reduced renal blood flow and increased renal vascular resistance [13], which are factors directly related with the impairment of renal function. However, the glomerular filtration rate, which is considered the best parameter of renal function [14], it has been poorly evaluated in the murine model of spontaneous hypercholesterolemia. In the present study, the glomerular filtration rate (GFR) was estimated by the creatinine clearance method. The main finding of our study is that GFR was markedly impaired in the ApoE mice already at 2-month-old. This finding corroborates the idea that renal damage promoted by hypercholesterolemia could occurs independently of renal artery stenosis [15], since young animals do not show atherosclerotic plaques yet [16,17].

Although no changes in glomerular tuft area were found, in agreement with no differences in glomerular volume in ApoE mice [18], renal histology analysis showed a marked mesangial expansion in young ApoE mouse, which could be favored by the increased leukocyte migration through glomerular capillaries [19] in response to increased expression of adhesion molecules caused by oxidized cholesterol [20] and thus contributing to the loss of renal function. A plausible explanation for the effects of mesangial cells on the filtration rate is that they present contractile properties in response to endogenous vasoactive agents [21]. The mesangial expansion also contributes to the extracellular matrix expansion, which in turn could affect the filtration rate by reducing the glomerular permeability, as indicated by other studies [21]. However, we cannot rule out the participation of other hypercholesterolemia-induced factors contributing to the renal dysfunction, including reduced nitric oxide bioavailability, exacerbation of the renin-angiotensin system and increased oxidative stress [15,22,23]. In accordance with this data, we observed marked renal fibrosis in young hypercholesterolemic animals. Increase in kidney collagen deposition is usual pathologic observation in a variety of many renal diseases, suggesting a final common pathway in progressive renal injury [24]. Dyslipidemia can cause renal fibrosis due to increase in extracellular matrix deposition and reduced matrix degradation [25]. The increase in collagen synthesis and activation of pro-inflammatory pathways by oxidized low density lipoprotein (LDL) could be responsible for increased matrix deposition. It has already been shown that renal tubular cells can express scavenger receptors, which are responsible for oxidized LDL uptake, acting as a bridge between hyperlipidemia, oxidative stress and renal fibrosis [26].

Once the rising number and proportion of aged individuals is a global demographic trend [27], the use of advanced age kidney donors appears as an alternative to reduce the number of patients in the waiting list for a transplant, but the implications of this procedure are not well established. The aging process is known to cause kidney dysfunction, as has been observed in human beings [14] and experimental animals [28,29], but not yet reported in hypercholesterolemic ApoE mouse. Our data show that the impairment of renal function was worsened in 8-month-old ApoE mice and onset in age-matched C57 control animals. This result indicates that aging is an important and independent risk factor for the renal disease. This is corroborated by the finding that the reduction of renal function observed in the aging process is associated with a previous renal vasoconstriction, which leads to a reduced renal blood flow and consequently decrease in glomerular filtration rate [30,31]. There are also indications that temporal renal damage is accompanied by an increase in oxidative stress and consequently reduced bioavailability of vasodilator substances [32], which could explain, at least in part, our findings.

Increased uremia is a hallmark of renal failure [33]. Our data show an increased uremia in ApoE mice without additive effect of aging, in agreement with others [34]. Surprisingly, even though C57 mice presented a time-dependent reduction in glomerular filtration rate, this condition was not accompanied by an increase in uremia. A possible explanation for this finding could be a temporal reduction in the expression of urea transporters in the kidney [35]. This change could reduce the urea reabsorption, with consequent increased excretion of urea in the urine, which could not be true for the ApoE mouse that already present high uremia at young age.

The role of nitric oxide (NO) in the development of renal dysfunction due to aging cannot be ruled out, since this vasodilator agent plays an important role in glomerular hemodynamic [36]. Aging process promoted a reduction in nNOS expression in both mice strains. It has already been reported that reduced nNOS expression and NO production are directed related to renal injury in a time-dependent manner in Sprague-Dawley rats [37]. In addition, NO is also known as an anti-growth agent that can inhibit mesangial cell proliferation and therefore extracellular matrix production [38]. This could be related to both age-dependent mesangial expansion and reduction in glomerular filtration rate, as observed in adult C57 and ApoE animals in the present study.

## Conclusion

Considering that the general health of the donor and the functional renal reserve should determine the upper age limit for an ideal kidney donor [39], this study emphasizes the importance of treating dyslipidemic patients at an early phase, to avoid the detrimental effects of high cholesterol levels on renal physiology. It seems that, even in the absence of established atherosclerotic plaques, hypercholesterolemia can have deleterious effects on renal function, probably due to mesangial expansion and tubulointersticial fibrosis, as observed in young animals.

## Methods

### Animals

Experiments were performed using male young (2-month-old) and adult (8-month-old) Apolipoprotein E-deficient mice (ApoE; n = 13) and age-matched wild-types C57BL/6 (C57; n = 15). Animals were obtained from the breeding facility of the Transgenes and Cardiovascular Control Laboratory at the Federal University of Espirito Santo. Mice were fed a regular mouse chow (Labina^®^) and water *ad libitum*, housed in individual cages, in a temperature (22°C) and humidity (50%) controlled room, under a 12 hours dark/light cycle. All experimental procedures were done in accordance with the National Institute of Health (NIH) Guide for the Care and Use of Laboratory Animals and the protocols were previously approved by the Institutional Ethics Committee for Use of Animals (CEUA, Protocol 003/2009).

### Metabolic and Biochemical Parameters

Young and adult mice were housed individually in metabolic cages to 24-hour urine volume determination and measurements of chow and water intake. After that, animals were euthanized with an overdose of thiopental (Cristalia, Sao Paulo, Brazil, 200 mg/kg, *i.p*.) and had their blood collected for creatinine, cholesterol and urea measurements. Tissues were perfused with cold phosphate-buffered saline (PBS, pH 7.4, 0.1 M) through the left ventricle and one kidney was removed for Western Blot analysis. The remaining kidney was fixed in paraformaldehyde 4% in PBS to histological evaluation.

Serum cholesterol, urea and creatinine and urine creatinine were measured using colorimetric kits (Bioclin^®^, Belo Horizonte, Brazil). Creatinine clearance was calculated using plasma and urine creatinine concentrations and urine flow through the standardized formula.

### Kidney Histology

After perfusion, kidneys were fixed with 4% paraformaldehyde in PBS and processed for morphometric and histological analyses. Samples were dehydrated with increased alcohol concentrations and mounted in paraffin blocks. After that, kidneys were sliced in a microtome (AO-820, American Optical, New York, USA) in 10 μm-thick sections, stained with hematoxylin-eosin and analyzed at a 40 × magnification. Images were captured with a color video camera (VKC150, Hitachi, Tokyo, Japan) connected to a microscope (AX70, Olympus, Center Valley, PA, USA). All morphometric and histological analysis were performed in a single-blind manner.

The total number of glomeruli was obtained by counting all glomeruli in 10 sections separated from each other for about 200 μm to avoid counting the same glomerulus twice. The mean glomerular tuft area and Bowman's capsule area of each kidney were obtained by calculating the mean value of 100 glomeruli individual areas measured using the Image J program (1.33u, Public Domain).

Periodic Acid Schiff (PAS) staining was performed to evaluate the presence of mesangial expansion and Masson's Trichrome staining was used to tubulointersticial fibrosis quantification. A total of 30 glomeruli were used to calculate the percentage of stained area of each kidney using the Image J program.

### nNOS Expression

Expression of neuronal isoform of the enzyme nitric oxide synthase (nNOS) was performed by Western Blot, as previously described [40]. Briefly, after perfusion, kidneys were homogenized with a protease inhibitor cocktail (Product #P2714, Sigma Aldrich, St Louis, USA) and centrifuged at 11,000 rpm for 20 minutes at 4°C. The supernatant was collected and protein concentration was determined by Bradford method [41]. Protein samples were subjected to electrophoresis in polyacrylamide gel with sodium dodecyl-sulphate (SDS-PAGE) and electroblotting into a nitrocellulose membrane, which was then blocked with 10% skimmed milk in 0.1% PBS with Tween-20, for 60 minutes at room temperature. After that, membranes were incubated with a polyclonal antibody for the nNOS (Santa Cruz Biotechnology, St Cruz, USA) at 1:250 dilution overnight at 4°C, following by incubation with horseradish peroxidase-labeled secondary anti-rabbit antibody (GE Healthcare, Product #RPN2108, Buckinghamshire, UK) at 1:3000 dilution. Bands were detected by enhanced chemiluminescence in x-ray film, and band intensity was measured by densitometry using the Image J program (1.33u, Public Domain). Glyceraldehyde 3-phosphate dehydrogenase (GAPDH) expression was used as a housekeeping control.

### Statistical analysis

Data are presented as mean ± SEM. Statistical analysis was performed using 2-way ANOVA, followed by the Fisher's *post hoc *test. The significance level was set at p < 0.05.

## Competing interests

The authors declare that they have no competing interests.

## Authors' contributions

CMB carried out the animal experiments, analysis of the data and drafted the manuscript. MZTO performed the kidney histology experiments. TMCP participated in the design of the study and performed Western Blotting analysis. NFS participated in renal histological evaluation. ALG participated in the design and co-supervision of the study, performed the statistical analysis and participated in manuscript preparation. ECV participated in the design and co-supervision of the study and in the critical revision of the manuscript. SSM conceived the study, participated in its design and supervision and in the critical revision of the manuscript. All authors read and approved the final manuscript.
